# Facing the Emotional Barriers to Colorectal Cancer Screening. The Roles of Reappraisal and Situation Selection

**DOI:** 10.1007/s12529-024-10284-4

**Published:** 2024-04-18

**Authors:** Giulia Scaglioni, Miriam Capasso, Marcella Bianchi, Daniela Caso, Nicoletta Cavazza

**Affiliations:** 1 Department of Communication and Economics, University of Modena and Reggio Emilia, Reggio Emilia, Italy; 2 Department of Humanities, University of Naples Federico II, Naples, Italy

**Keywords:** Health behaviors, Emotional regulation, Disgust, Embarrassment, Fear, Early detection of cancer

## Abstract

**Background:**

Disgust, embarrassment, and fear can hinder the attendance of colorectal cancer (CRC) screening. However, individuals can respond to these emotions differently. The present study tested whether reappraising a negative stimulus versus avoiding a negative stimulus is associated with age; whether these two emotion regulation strategies (reappraisal and situation selection) moderate the effects of disgust, embarrassment and fear on CRC screening intention; and the efficacy of a message based on participants’ preferred emotion regulation strategy.

**Methods:**

We recruited 483 Italian participants (aged 40–84 years) through snowball sampling. Participants were randomly assigned to one of four conditions differing for a message promoting CRC screening with an affective lever, a cognitive lever, both levers or none. Key variables included emotion regulation strategies, emotional barriers and intention to get screened.

**Results:**

The preference for reappraisal over situation selection increased with age. Reappraisal neutralized the effect of disgust on CRC screening intention. The combined message with both affective and cognitive levers increased CRC screening intention (*b* = 0.27, β = 0.11, *SE* = 0.13 *p* = .049), whereas reading the message based only on the affective (*b* = 0.16, β = 0.06, *SE* = 0.14 *p* = .258) or the cognitive (*b* = 0.22, β = 0.09, *SE* = 0.14 *p* = .107) lever was not effective.

**Conclusions:**

Communication campaigns should support the activation of a reappraisal strategy of emotion control, and messages promoting CRC screening should highlight both the instrumental (i.e., early detection) and affective (i.e., peace of mind) benefits of attendance.

**Supplementary Information:**

The online version contains supplementary material available at 10.1007/s12529-024-10284-4.

## Introduction

Choosing to attend colorectal cancer (CRC) screening might seem an easy decision, because screening can reduce the incidence of this malignancy by 25.5% and its mortality by 52.4% [1]. However, in Italy, where the following study was conducted, less than one in two people in the target population attend CRC screening [2]. Most of the Italian local CRC screening programs offer a free of charge biennial fecal collection test, and a free of charge colonoscopy if this test reveals a positive result (i.e., the presence of blood in the stool [3]).

Given the importance of early detection, research is needed to understand which factors foster or hinder CRC screening exams attendance, and how these factors can be addressed using persuasive communication. For example, prior studies found that positive attitudes toward CRC screening, perceiving the test as reassuring, and being generally oriented toward health prevention behaviors can be CRC screening intention facilitators (for reviews, see [4, 5]). Also age seems to be associated with screening attendance, but prior findings have been highly mixed in this regard (for a review, see [6]).

Much evidence confirmed that CRC screening avoidance can be explained, at least partially, by its negative perception: colonic inspections (e.g., colonoscopy) and fecal collection tests (i.e., fecal occult blood and fecal immunochemical tests) are considered disgusting (for a review, see [7]), embarrassing [8], and scary [9, 10]. However, the impact of emotional barriers (i.e., disgust, embarrassment, and fear) on behavioral intention and subsequent actions can vary as a function of individual differences (e.g., trait disgust [11]).

For instance, the emotion regulation strategies that people activate to cope with their affective states might spill over in the CRC screening context, also in relation to people’s age [12]. Thus, the first aim of the present study was testing whether regulation strategies can neutralize the effects of negative emotions on screening intention, also considering the target’s life stage.

### Emotion Regulation Strategies

Emotion regulation strategies influence which emotions are experienced (or anticipated) and when, their intensity and expression, as well as their effects on behavior [13, 14]. Two emotion regulation strategies might be particularly important in the CRC screening context: situation selection and cognitive change. Situation selection implies selecting to approach or avoid a given experience according to its anticipated emotional outcome [15]; cognitive change, also called reappraisal [15], modifies the mental representation of a situation and, subsequently, its emotional impact [16].

People who tend to reappraise negative stimuli might move their attention from the *emotional barriers* associated with the screening procedure and its potential outcomes [10] to its benefits [17, 18]. On the other hand, people who tend to downregulate negative affective states with avoidance (i.e., situation selection) might be more likely to delay unpleasant medical procedures such as CRC screening [15].

The preference for situation selection can increase with age [19–21]. According to the socioemotional selectivity theory [12], older adults’ preference for situation selection might be due to the perception of a limited future time horizon, which motivates older adults to engage only in pleasant situations [22]. On the other hand, younger adults’ perception of a longer time horizon encourages them to have multiple and long-term goals, and motivates them to sacrifice their current emotional well-being for this goal pursuit [19], which is consistent with the reappraisal strategy. Thus, older adults might be less prone to attend CRC screening. In addition, the age-related preference for a specific emotion regulation strategy has consequences on how information is processed and which type of communication can be effective: older adults dedicate more attention to emotionally (vs. cognitively) relevant information [12, 23], whereas younger adults tend to prefer rational appeals [22, 23]. The communication promoting CRC screening should acknowledge these differences.

Our second aim was testing the effectiveness of messages that included emotionally relevant information, rational appeals, or both, compared to a control message (no affective or cognitive arguments), and investigating whether the effects of these messages varied as a function of the preference over reappraisal or situation selection. Indeed, portraying CRC screening in an emotionally positive light (i.e., as an experience that brings peace of mind [24]), might be more effective for those participants who prefer situation selection rather than reappraisal, whereas a detached argument about the importance of getting screened [25] might be more effective for those participants who prefer reappraisal over situation selection.

### The Present Study

In line with the framework of the socioemotional selectivity theory [12], we expected that the preference for situation selection over reappraisal would increase with age (hypothesis 1).

As reappraisal might lead to attending CRC screening *despite* the emotional barriers associated with its procedure and outcomes, whereas situation selection may cause CRC screening avoidance *because of* these emotional barriers, we expected a moderation effect of emotional regulation strategy on the association between disgust (hypothesis 2a), embarrassment (hypothesis 2b) and fear (hypothesis 2c) and CRC screening intention, with people who prefer reappraisal being less affected by these emotional barriers.

Since matching persuasive messages to the target’s characteristics can boost screening attendance [26, 27], and considering the effects of preferring a specific emotion regulation strategy on information processing [14, 22, 23], we expected to find an interaction between the type of lever included in the message and participants’ preferred emotion regulation strategy: the cognitive lever (vs. the affective or no lever) would be associated with a higher intention to attend CRC screening for receivers who prefer reappraisal (hypothesis 3a); in comparison, the affective lever (vs. the cognitive or no lever) would be associated with a higher intention to attend CRC screening for those who prefer situation selection (hypothesis 3b). In other words, we expected the preference over an emotion regulation strategy to moderate the effects of the messages on CRC screening intention (two-way interaction).

Finally, we expected that giving salience to the cognitive and affective benefits of CRC screening in the message would improve the message efficacy. In other words, we expected a main positive effect of using the affective (hypothesis 4a), the cognitive (hypothesis 4b), or both levers (hypothesis 4c) on CRC screening intention (vs. the control message without any affective or cognitive levers). Orientation toward prevention (i.e., the tendency of undergoing medical examinations) was controlled in the analyses because it can impact screening behaviors [28].

Hypotheses 1 to 2c relate to our first aim (i.e., investigating the role of emotion regulation strategies in the CRC screening context), while hypotheses 3a to 4c pertain to our second aim (i.e., studying the promotion of CRC screening, and in particular the matching effects of affective and cognitive levers with emotion regulation strategies).

## Method

### Participants

We aimed to include people over 40 years of age (i.e., near the age of the CRC screening target), living in Italy and with no previous history of CRC. We included participants under and over the recommended screening age (i.e., 50−69 years old) to compare older and younger adults, and because these participants might have attended screening with private care, might be contemplating screening for CRC [29] or have attended screening in the past.

Participants were recruited through snowball sampling using advertisements on social media (please see Electronic Supplementary Material 1 for the study advertisement details). To estimate the minimum sample size, we conducted an a priori power analysis [30]. Following Capasso et al. [31], who tested the efficacy of several messages on COVID-19 vaccination intention, we considered a small-sized effect (η^2^ = 0.04), α = 0.05 and power = 0.80. This analysis indicated a minimum sample size of 100 participants per group (i.e., 400 participants) to detect differences between each experimental condition and the control group.

**Fig. 1:**
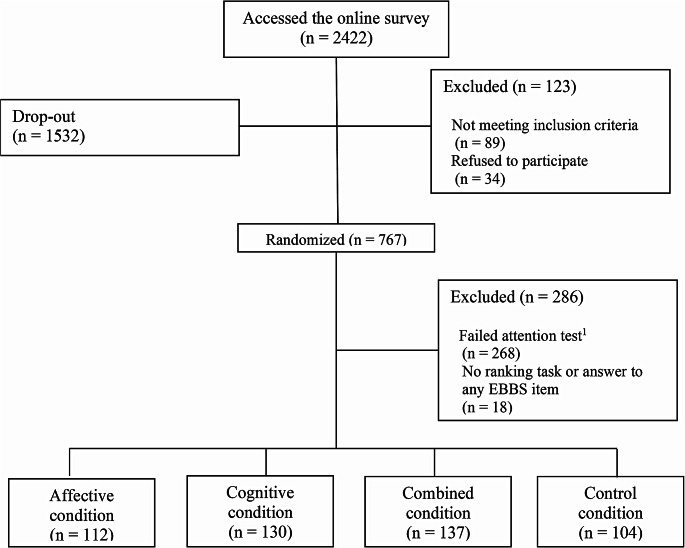
Participants’ recruitment, drop-outs, exclusions and final sample. *Note*^1^ The attention test asked participants which words they read in the message (*relieved and peaceful; useful and important; effective and happy, none of the above*)

A total of 2422 people accessed the online questionnaire over three months (18 May 2022–31 July 2022). After drop-outs and exclusions (Fig. 1), the final sample consisted of 483 participants (78% women) who were randomly assigned to one of four experimental conditions. As Capasso et al. [31] did not consider interactions in their power analysis, we controlled with G*Power [30] the sample we obtained. Considering seven interactions and 15 total predictors (the interactions plus the single variables and three control variables), with our 483 participants we could detect at least a small interaction ( *f*^2^ = 0.04).

Participants’ age ranged from 40 to 84 years old; 53.4% of participants had a senior high-school leaving qualification, and 30.4% had a university-level degree; 58.8% of the participants were employed, and 19.5% were unemployed or retired; less than half of the sample (41.6%) had received at least one formal invitation to screen (e.g., a screening invitation letter from the local health care authority) and, among these, 84.6% reported to having attended CRC screening at least once. Respondents in the four experimental groups did not differ in age, gender, education, employment or screening experience (Table 1).

**Table 1:** Characteristics of participants in each condition and between-group differences

	Affective message	Cognitive message	Combined message	Control message	Between-group differences
	n (%)	n (%)	n (%)	n (%)	
Gender					χ^2^ (3) = 4.76, *p* = .190
Female	96 (86%)	99 (76%)	104 (76%)	79 (76%)	
Male	16 (14%)	31 (24%)	33 (24%)	25 (24%)	
Education					χ^2^ (9) = 8.50, *p* = .484
Compulsory education	3 (3%)	14 (11%)	12 (9%)	6 (6%)	
High school	58 (52%)	66 (51%)	76 (55%)	58 (56%)	
University	39 (35%)	39 (30%)	37 (27%)	32 (31%)	
Post-graduate education	12 (10%)	11 (8%)	12 (9%)	8 (7%)	
Employment					χ^2^ (12) = 12.53, *p* = .404
Employee	67 (60%)	81 (62%)	75 (55%)	61 (59%)	
Self-employed	22 (20%)	17 (13%)	20 (15%)	10 (10%)	
Unemployed	5 (5%)	8 (6%)	13 (9%)	14 (13%)	
Retired	12 (10%)	15 (12%)	16 (12%)	11 (11%)	
Other	6 (5%)	9 (7%)	13 (9%)	8 (7%)	
CRC screening experience					χ^2^ (6) = 5.25, *p* = .512
Refused to screen	3 (3%)	8 (6%)	12 (9%)	8 (7%)	
Never invited	64 (57%)	66 (51%)	65 (48%)	54 (52%)	
Attended screening	45 (40%)	56 (43%)	59 (43%)	42 (41%)	

*Note*  Participants’ mean age varied from 50 years old (*SD* = 7.86) in the affective condition to 52 years old (*SD* = 8.63) in the cognitive condition. The difference between the four conditions was not statistically significant, *F* (3, 432) = 0.70, *p* = .553

### Procedure

This study was approved by University of Parma Research Ethics Board (Protocol number 0107278) and pre-registered before data collection on AsPredicted (www.aspredicted.org; #96773). The final work presented several differences from the initial project. These differences are reported in Electronic Supplementary Material 2. The experimental material and dataset are available at https://osf.io/59fsz/?view_only=6a76485f25aa43399fa2ac0be8001881.

We asked participants to fill in an online questionnaire anonymously. Each participant was randomly assigned to one of the four conditions through the survey platform. The conditions differed only for the message shown. Participants were blind to the hypotheses and experimental conditions. Messages were presented in a graphic format inspired by images usually shared on the official Facebook page of the Italian Ministry of Health. Indeed, graphic messages can enhance the understanding of the information conveyed within health messages (e.g., [32]). because they are easy to comprehend and emotionally engaging [33]. The intervention was designed based on a recent study [31] and followed the guidelines for affect-based interventions [34]. The messages and the English translation are reported in Electronic Supplementary Material 3.

The survey started with a general description of the experiment, and participants were asked to give informed consent to participation and data treatment. The first questions established whether participants met our inclusion criteria, recorded sociodemographic data, and assessed participants’ orientation toward prevention (see below). Then we assessed participants’ preference for reappraisal or situation selection with a ranking task. Afterwards, we briefly explained what CRC screening is, measured past attendance, and exposed participants to an experimental stimulus for a total lapse of approximately 10 s. Specifically, participants read one of the four manipulated messages.

Participants’ attention was checked with an item presented just after the stimulus exposure, asking participants to remember which words were included in the message. Then, participants were invited to imagine they received an invitation to get screened for CRC (regardless of age) containing the previously-read message. Consequently, they rated their emotions toward bowel screening and their CRC screening intention. Finally, the debriefing explained the purpose of the study and, to guarantee equal treatment, participants could read the other messages.

### Measures

#### Emotion Regulation Strategies

We asked participants to rank six sentences describing behaviors consistent with *reappraisal* or *situation selection* from 1 (the most representative of your typical behavior) to 6 (the least representative of your typical behavior). Sentences were presented in random order. Three sentences operationalizing reappraisal (e.g., “I control my emotions by changing the way I think about the situation I’m in”) were derived from the Emotion Regulation Questionnaire [35] and were adapted for the Italian context by Balzarotti et al. [36]. The other three sentences concerned situation selection (i.e., “I shy away from situations that might upset me”) and were adapted from Webb et al. [37]. Following the procedure by Osborne et al. [38], reappraisal ranks were converted to scores so that ranks 1, 2, 3, 4, 5 and 6 received corresponding scores of 6, 5, 4, 3, 2, and 1. A mean was obtained from the three items of the reappraisal strategy, being the score relative to the situation selection complementary (i.e., the situation selection score is the exact opposite of the reappraisal score). Therefore, a higher score indicates a greater preference for reappraisal and a lower score indicates a greater preference for situation selection.

#### Emotional Barriers Associated with CRC Screening

Disgust, embarrassment, and fear were measured using a version of the Emotional Barriers to Bowel Screening scale [10, 39] that was translated and reduced for the Italian CRC screening context by Scaglioni and Cavazza [40]. Participants rated the likelihood of experiencing each emotion on a scale from 1 (not likely at all) to 5 (very likely). Four items measured *faecal disgust* (α = 0.90, e.g., “Collecting a sample of my faeces on a stick would be disgusting”), three measured *embarrassment* (α = 0.89, e.g., “Delivering the sample of faeces would be embarrassing”), and three measured *fear of the outcome* (α = 0.71, e.g., “I would worry that this test might find something wrong with me”). Higher scores corresponded to a greater likelihood of experiencing negative emotions elicited by CRC screening. Mean scores were calculated for each subscale.

#### Intention to get Screened

We measured CRC screening intention in two different ways: one item asked participants when they would get screened after reading the message (*never*, *within a week*, *a month*, *a year* or *willing to get screened but procrastinating*), and five items assessed, on a 5-point Likert scale, their willingness to perform every screening step (collecting the kit, collecting the sample, storing the collected sample, taking the collected sample to be analyzed, undergoing a colonoscopy in case of a positive test result). Each item asked participants to answer regardless of their age, imaging that the local health authority invited them to get screened.

#### Control Variables

Orientation toward prevention was assessed with two items: “In general, how careful would you say you are in the prevention of possible diseases?” and “In general, how interested would you say you are in disease prevention programs?”. Scores were expressed on a scale from 1 (not much careful/interested) to 5 (very careful/interested*)*. A mean score was calculated (*r* = .56, *p* <.001). *Screening experience* was assessed with two items asking the participant whether they had ever received a formal invitation to screen and whether they had ever attended. A single score was calculated with non-attendees (“−1”, people who received a formal invitation to get screened but did not), never-invited (“0”, people who were never invited to get screened), and attendees (“1”, people who attended CRC screening at least once).

## Results

### Preliminary Analyses

The single item assessing screening delay after receiving an invitation to screen (see *Measures)* was less affected by ceiling effects (*M* = 4.04, *SD* = 1.07, skewness = −1.00) than the five items assessing the intention to engage in every screening step (*M* ≥ 4.36, *SD* ≤ 0.93, skewness ≤ −1.49). Furthermore, the former measure of intention was strongly correlated to the averaged measure of those five intention items (*r* = .52, *p* ≤.001). Therefore, establishing screening intention with the amount of time participants would wait to get screened was considered a reliable measure and adopted as the only outcome.

Table 2 reports descriptive statistics and correlations. CRC screening was mainly associated with the fear toward a potential cancer diagnosis, followed by a moderate disgust toward dealing with feces and, lastly, embarrassment.

**Table 2:** Means, Standard Deviations, and Correlations between variables

	M (SD)	1	2	3	4	5	6	7	8	9
1. Age	51.09 (8.33)									
2. Screening Experience	−	0.50***								
3. Orientation toward prevention	3.84 (0.95)	0.26***	0.32***							
4. Emotion regulation strategy	3.55 (0.94)	0.12**	0.06	0.01						
5. Cognitive lever	−	0.06	0.01	0.04	0.02					
6. Affective lever	−	−0.02	0.01	0.03	0.02	−0.01				
7. Disgust	1.44 (0.89)	−0.06	−0.18***	−0.12**	−0.02	0.03	−0.03			
8. Embarrassment	1.36 (0.85)	−0.08	−0.23***	−0.14**	0.01	−0.05	0.01	0.62***		
9. Fear	2.32 (1.01)	−0.03	−0.15***	−0.05	−0.08	−0.05	−0.02	0.31***	0.29***	
10. Screening intention	4.04 (1.07)	0.10*	0.19***	0.34***	0.07	0.09	0.06	−0.19***	−0.15***	−0.05

Note * p ≤.05, ** *p* ≤.01, *** *p* ≤.001

Respondents’ gender (*t* (150.93) = 0.14, *p* = .883), education (*r* = −.09, *p* = .057) and employment (*F* (4, 475) = 2.08, *p* = .083) did not affect the outcome and were not further considered. Respondents’ age, prior CRC screening experience and orientation toward prevention were significantly associated with CRC screening intention and, as such, were considered covariates in the subsequent moderated regression analysis (Table 2).

### Main Analyses

We first investigated whether preferences for reappraisal and situation selection were related to participants’ age. We expected a negative correlation between the emotion regulation strategy score and age (hypothesis 1), meaning that older adults prefer situation selection, whereas younger adults prefer reappraisal. We found the opposite, as age and the reappraisal score were positively correlated (Table 2).

**Fig. 2:**
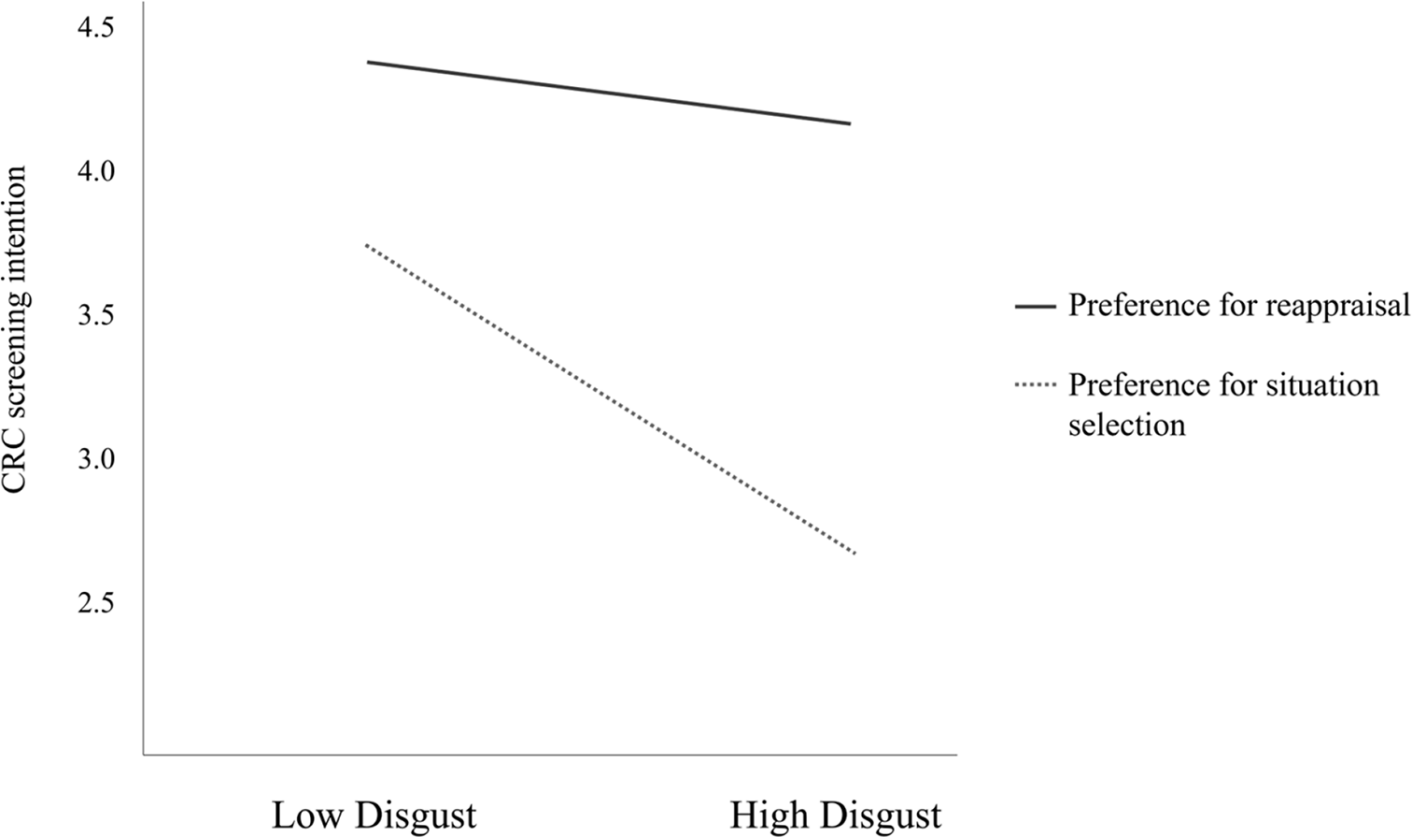
Single slope analyses. Effect of disgust on colorectal cancer screening intention as a function of preferring situation selection versus reappraisal

Then, to test whether reappraisal reduced the impact of disgust, embarrassment and fear on CRC screening intention (hypotheses 2a−2c), whether this emotion regulation strategy interacted with being exposed to affective and/or cognitive arguments promoting CRC screening (hypotheses 3a and 3b), and whether the messages had a main effect on CRC screening intention (hypotheses 4a−4c), net of respondents’ age, prior CRC screening experience and orientation toward prevention, we ran a moderated regression analysis. Focal predictors (i.e., disgust, embarrassment and fear) and the moderator (i.e., emotion regulation strategy) were mean-centered to facilitate results interpretation. Experimental conditions were treated as categorical variables, with the control message as reference category. The analysis yielded a main negative effect of disgust (*b* = −0.28, β = −0.23, *SE* = 0.09, *p* = .001), and this emotion significantly interacted with preferring a reappraisal regulation strategy (*b* = 0.21, β = 0.14, *SE* = 0.10, *p* =.038), thus confirming hypothesis 2a. Single slopes analyses (Fig. 2) showed that disgust was a screening deterrent only for those participants who preferred situation selection (*b* = −0.48, *SE* = 0.14, *p* <.001) rather than reappraisal (*b* = −0.09, *SE* = 0.12, *p* = .451).

In comparison, fear and embarrassment were not associated with CRC screening intention (fear, *b* = 0.01, β = 0.01, *SE* = 0.05 *p* =.849; embarrassment, *b* = 0.08, β = 0.06, *SE* = 0.10, *p* = .419), and did not interact with the emotion regulation strategy (fear*reappraisal, *b* = −0.07, β = −0.06, *SE* = 0.05 *p* =.189; embarrassment*reappraisal, *b* = −0.14, β = −0.09, *SE* = 0.11, *p* =.194). Hence, our findings did not support hypotheses 2b and 2c.

**Fig. 3:**
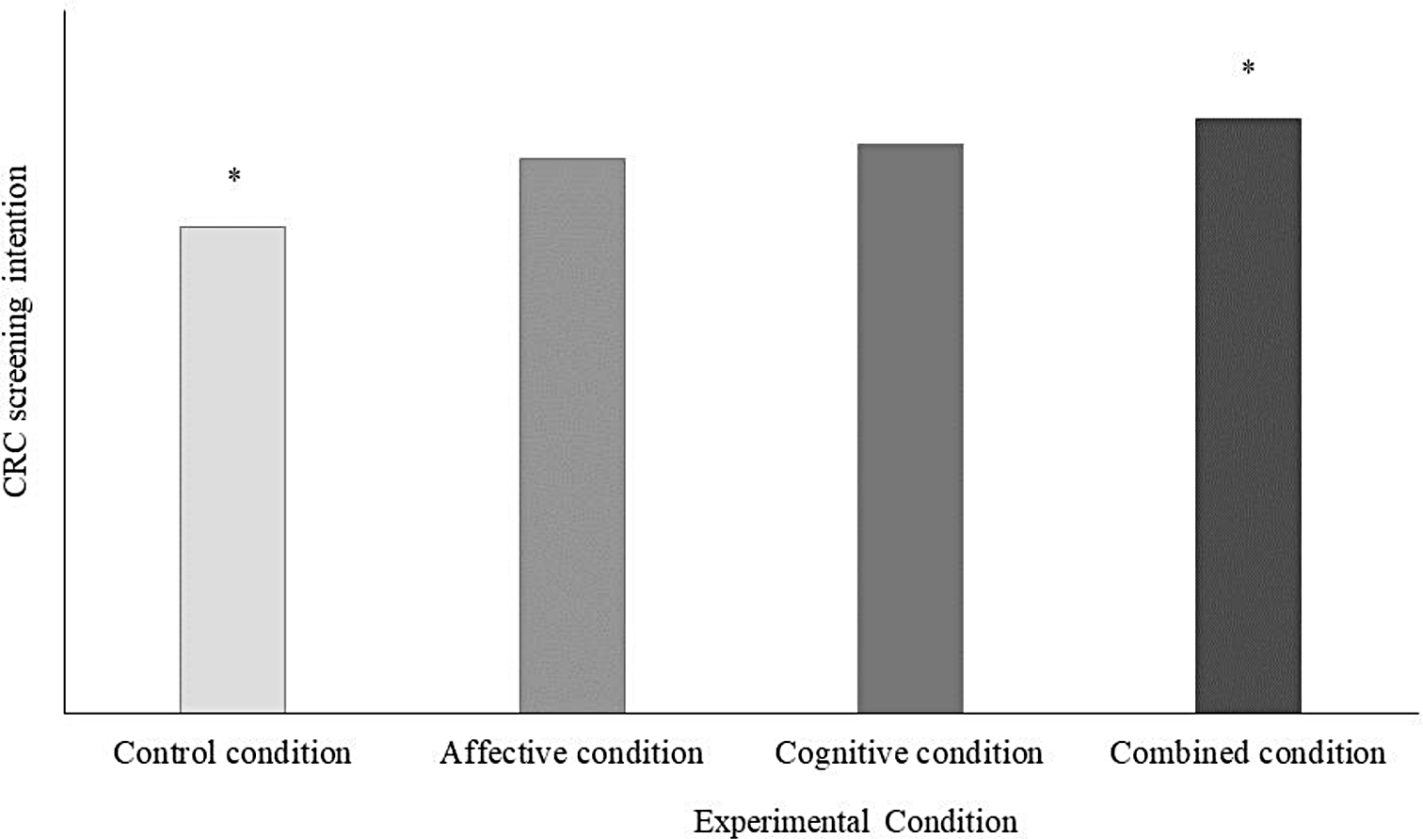
Between-groups differences in CRC screening intention. *Note* * Significantly different (*p* <.05)

No matching effect emerged from the interactions between preference for reappraisal and experimental conditions (affective condition*reappraisal, *b* = 0.03, β = 0.01, *SE* = 0.15 *p* =.832; cognitive condition*reappraisal, *b* = −0.03, β = −0.01, *SE* = 0.14 *p* = .832; combined condition*reappraisal, *b* = 0.03, β = 0.02, *SE* = 0.14 *p* =.818). Thus, we rejected hypotheses 3a and 3b. Finally, the affective (*b* = 0.16, β = 0.06, *SE* = 0.14 *p* =.258) and cognitive (*b* = 0.22, β = 0.09, *SE* = 0.14 *p* =.107) messages did not boost CRC screening intention, but we found a main positive effect of reading the combined message (*b* = 0.27, β = 0.11, *SE* = 0.13 *p* =.049) versus the control message. Therefore, we rejected hypotheses 4a and 4b, but our findings supported hypothesis 4c (Fig. 3): those participants who read the combined message expressed a greater CRC screening intention (*M* = 4.15, *SD* = 1.07) than those assigned to the control condition, who read the message with no levers (*M* = 3.85, *SD* = 1.15). In contrast, reading the message based only on the affective (*M* = 4.03, *SD* = 1.06) or the cognitive lever (*M* = 4.08, *SD* = 1.01) did not significantly boost participants’ screening intention compared to control condition.

It should be noted that excluding individuals not eligible for screening (as well as those eligible) from the analysis makes the effect of the combined message not significant. However, in these cases the sample is too small to detect the observed effect.

## Discussion

The present study aimed to investigate the role of two emotion regulation strategies, situation selection and reappraisal, in the CRC screening context. The research examined whether preferring one of the two strategies depends on people’s age, whether a tendency to prefer reappraisal over situation selection might help individuals to cope with emotional barriers to CRC screening and, finally, we investigated the potential implications of emotion regulation strategies for designing effective persuasive communication of CRC screening.

We observed age-related differences in the preference for one strategy over the other but in the opposite direction of what was expected (hypotheses 1): older age was associated with an increased preference for reappraisal. Nevertheless, it should be noted that the correlation between age and the emotion regulation score can be interpreted as a small effect [41].

Although our findings are not in line with the motivational principles of the socioemotional selectivity theory (i.e., younger adults are expected to be more motivated to reappraise a situation in exchange of enduring benefits than older adults [12]), these results can be explained with the age-related positivity and negativity effect: some studies showed that younger adults tend to focus their attention on negative emotions (which might favor situation selection), whereas older adults tend to focus on the positive aspects of a situation (which can help them to adopt a reappraisal emotion regulation strategy) [43–44]. A similar explanation might be the different reactiveness to stressors of older and younger adults—younger adults are more affected by negative emotional inputs than older adults [45, 46].

After examining a potential determinant of the preference for reappraisal or situational selection, we analyzed how these regulation strategies may spill over in the CRC screening context. Our findings showed that disgust is a significant emotional deterrent to CRC screening intention, but only for those participants who preferred situation selection (i.e., approaching or avoiding a situation according to its emotional outcome). On the contrary, the effect of disgust on CRC screening intention was neutralized by adopting a reappraisal regulation strategy, that is focusing on the silver linings of potentially negative situations. These results were partially in line with our expectations, as they supported hypothesis 2a but not hypotheses 2b and 2c: we expected to find the same interaction for embarrassment and fear; however, these emotions were not associated with CRC screening intention in the regression model.

There was also no interaction between the emotion regulation strategy and the experimental messages (hypotheses 3a−4b not supported). Regardless of the preferred emotion regulation strategy, only the participants who read the combined message (i.e., the message including references to both positive anticipated affects and cognitive attitudes toward CRC screening) were more willing to get screened than the participants reading the control message (i.e., the message with no references to levers). Thus, in line with prior findings [31], the present study showed that messages targeting only anticipated affects or cognitive attitudes could not increase the intention of engaging in a health behavior. In contrast, emphasizing both the instrumental (e.g., CRC screening is useful) and the affective (e.g., attending CRC screening is relieving) benefits of CRC screening represents a further step that makes a message more persuasive than levering only affective or cognitive arguments [31].

### Limitations

We acknowledge that this work may be affected by several weaknesses, and its findings should be interpreted cautiously. First, we relied on self-reported measures and failed to include actual behavioral outcomes. We believe that our choice was justified because (I) screening intention is strongly associated with actual behavior [47]; (II) disgust impacts to the same extent on CRC screening intention and CRC screening attendance [7]; (III) self-reports are considered a “gold-standard” method to measure affect in terms of experiential feeling states [48]. Second, considering two emotion regulation strategies as alternatives could be another drawback of the present study, as different emotion regulation strategies can occur together [49]. Third, our participants formed a convenience sample recruited through social media and personal contacts; thus, the sample was not representative of the target population and overrepresented people with high educational attainment [50]. This might limit the external validity of the present findings. However, it should be noted that education and employment did not affect CRC screening intention; therefore, this overrepresentation might have little impact on our findings. More importantly, because of our recruitment strategy, this convenience sample is likely composed of people who were already favorable to CRC screening. More than half of the people who accessed the survey dropped out, which might indicate that the same barriers to CRC screening participation might have prevented study completion. Finally, we did not assess factors such as participants’ ethnicity, medical history and cancer familiarity, which might influence the present findings.

### Implications

Although the aforementioned limitations must be acknowledged, the present findings have several implications. To the best of our knowledge, no prior study investigated the impact of emotion regulation strategies in the CRC screening context. Thus, this work shed some light on the possibility of attenuating the effect of emotions in screening situations. Disgust, the main barrier to screening attendance, is weakened when people activate a reappraisal strategy of emotion regulation. This suggests a practical implication: besides trying to persuade people to get screened, preventive programs and public campaigns should point to reinforce the reappraisal strategy and address disgust. Messages about bowel screening should not portray heavily threatening experiences, because reappraisal is more likely to happen when emotional intensity is low rather than high [51–53]. In addition, as Wong et al. [54] found that text messages can effectively reinforce disgust reappraisal, we suggest that further studies should test the impact of messages explicitly recognizing that fecal collection or invasive exams can be perceived as disgusting, normalizing this reaction, and suggesting how can it be overcome.

Finding that older adults, and not younger adults, prefer reappraisal shows the importance of carefully considering social-psychological characteristics of the target audience before delivering an intervention. This also implies the need to increase the efforts to engage citizens who receive the CRC screening invitation letter for the first time because they are more vulnerable to emotional barriers than older invitees. Furthermore, the first invitation is crucial because screening behavior tends to crystallize and replicate in the long run [55]. Therefore, it is worth reserving demanding interventions for people who are invited for the first time (e.g., personal contacts with a health care specialist) to balance resources and benefits.

Finally, our findings confirm the importance of considering both cognitive and affective factors in designing more effective persuasive campaigns in the CRC screening context [47].

## Electronic Supplementary Material

Below is the link to the electronic supplementary material.

Supplementary Material 1

Supplementary Material 2

Supplementary Material 3
