# Correction: Facing the Emotional Barriers to Colorectal Cancer Screening. The Roles of Reappraisal and Situation Selection

**DOI:** 10.1007/s12529-024-10298-y

**Published:** 2024-05-23

**Authors:** Giulia Scaglioni, Miriam Capasso, Marcella Bianchi, Daniela Caso, Nicoletta Cavazza

**Affiliations:** 1 Department of Communication and Economics, University of Modena and Reggio Emilia, Reggio Emilia, Italy; 2 Department of Humanities, University of Naples Federico II, Naples, Italy

**Correction to: International Journal of Behavioral Medicine** 10.1007/s12529-024-10284-4

The original online version of this article was revised: Giulia Scaglioni's affiliation was updated to: Department of Communication and Economics, University of Modena and Reggio Emilia.

