# Supplementary material for: Facing the Emotional Barriers to Colorectal Cancer Screening. The Roles of Reappraisal and Situation Selection
Source: Int J Behav Med. 2024 Apr 18;32(4):505–14. doi: 10.1007/s12529-024-10284-4 (PMC12328489; doi:10.1007/s12529-024-10284-4)
Supplement: Supplementary file 1 — Supplementary Material 1 [file 12529_2024_10284_MOESM1_ESM.pdf]

ASPREDICTED
Wharton CREDIBILITY LAB
UNIVERSITY of PENNSYLVANIA

CONFIDENTIAL - FOR PEER-REVIEW ONLY

Age-related differences in facing emotional barriers to CRC screening (#96773)

Created: 05/11/2022 06:23 AM (PT)

This is an anonymized copy (without author names) of the pre-registration. It was created by the author(s) to use during peer-review.
A non-anonymized version (containing author names) should be made available by the authors when the work it supports is made public.

1) Have any data been collected for this study already?

No, no data have been collected for this study yet.

2) What's the main question being asked or hypothesis being tested in this study?

Among two emotion regulation strategies, we expect older adults to show a preference for situation selection (1a) and younger adults to show a preference for cognitive reappraisal (1b).
Age and the preferred emotion regulation strategy will moderate the effect of negative emotions on colorectal cancer screening intention (2).
We expect participants in the experimental conditions (vs. control) to report higher intention to screen (3).
We expect a matching effect between the emotion regulation strategy and a persuasive cognitive versus affective message: the cognitive message (vs. the other conditions) will be associated with a higher intention to screen for those who will express a preference for cognitive reappraisal (4a), while the affective message (vs. the other conditions) will be associated with a higher intention to screen for those who will express a preference for situation selection (4b).

3) Describe the key dependent variable(s) specifying how they will be measured.

The key dependent variable will be intention to screen for colorectal cancer.
We will measure this outcome in two different ways: one item asking participants when they would screen after reading the message (never, procrastination, within a week, a month, a year) and 5 items assessing, on a 5-point Likert scale, their willingness to perform every screening step (collecting the kit, collecting the sample, etc.).
We chose to adopt two different ways to measure screening intention because we want to explore ways to avoid ceiling effects.

4) How many and which conditions will participants be assigned to?

The experimental design is a 2 (cognitive cues in the message: present vs absent) x 2 (anticipated emotion cues: present vs absent) between participants.
Moreover, we will measure participants preferred strategy of emotion regulation, included in the design as a within variable.

5) Specify exactly which analyses you will conduct to examine the main question/hypothesis.

H1, H3, H4: ANOVA
H2: moderated regression analysis

6) Describe exactly how outliers will be defined and handled, and your precise rule(s) for excluding observations.

We will exclude participants who do not respond to enough items to allow our analyses (i.e., age, at least one item per emotion, emotion regulation strategy task, at least one intention item) who will not meet the inclusion criteria (aged 40-80, living in Italy, not having received any CRC diagnosis), who will not give their consent, and who will fail the manipulation check.

7) How many observations will be collected or what will determine sample size? No need to justify decision, but be precise about exactly how the number will be determined.

Using a small-sized effect (eta2 = 0.04), alpha = .05 and power = .80, the a priori power analysis indicates a minimum sample size of 100 per condition to detect differences between the control and each experimental condition. Thus, we will keep recruiting participants until the target number of 400 is achieved.

8) Anything else you would like to pre-register? (e.g., secondary analyses, variables collected for exploratory purposes, unusual analyses planned?)

For exploratory purposes, we will analyze the effect of the message on negative emotions, and we will test the effects of a combined cognitive plus affective message on screening intention.
