# Supplementary material for: Facing the Emotional Barriers to Colorectal Cancer Screening. The Roles of Reappraisal and Situation Selection
Source: Int J Behav Med. 2024 Apr 18;32(4):505–14. doi: 10.1007/s12529-024-10284-4 (PMC12328489; doi:10.1007/s12529-024-10284-4)
Supplement: Supplementary file 3 — Supplementary Material 3 [file 12529_2024_10284_MOESM3_ESM.docx]

# Electronic Supplementary Material 1

The advertisement stated:

“We are looking for participants!

Hi, we are conducting a research about the perception of colorectal cancer screening.

If you are older that 40 years old, help us!

Read a short message and answer to an anonymous survey.

Every participation counts!”

To access the survey, the advertisement included a QR code and a link.

# Electronic Supplementary Material 2

We applied the following differences from the initial pre-registered project for the sake of the paper's fluency or appropriateness:

1. The association between age and emotion regulation strategy was assessed with a correlation analysis in place of an ANOVA to avoid splitting a continuous variable.
2. Single ANOVAs to test our hypotheses were replaced with a single moderated regression analysis.
3. Age was removed as a moderator by the moderated regression analysis.

4. We do not report the effect of the message on negative emotions.

The originally planned analyses are now reported.

The one-way ANOVA comparing the effects of age group (younger vs. older than 50 years old) on the preference for reappraisal versus situation selection strategy revealed the opposite of what we expected: older adults showed a preference for cognitive reappraisal (*M* = 3.66, *SD* = .93) and younger adults showed a preference for situation selection (*M* = 3.41, *SD* = .94), *F*(1, 435) = 7.90, *p* = .005.

The 2 (cognitive cues in the message: present vs absent) x 2 (anticipated emotion cues: present vs absent) x 2 (preferred emotion regulation strategy: situation selection vs reappraisal) ANOVA found no main nor matching effect (Supplementary Table 1).

We originally expected age and the emotion regulation strategy to moderate the effect of disgust, embarrassment, and fear on colorectal cancer (CRC) screening intention. To test these hypotheses, we ran a moderated regression analysis for each emotion with disgust, embarrassment and fear as focal predictors, CRC screening intention as outcome, age group and emotion regulation strategy as moderators, net of respondents’ education, prior CRC screening experience and orientation towards prevention. The first moderation analysis, including disgust as focal predictor, yielded a main effect of this emotion (*b* = −2.36, *SE* = .84, *p* = .005) and age group (*b* = −1.51, *SE* = .76, *p* = .046) on the outcome. Emotion regulation strategy had no main effect on CRC screening intention (*b* = −0.47, *SE* = .35, *p* = .180). Disgust significantly interacted with both moderators, age group (*b* = 1.16, *SE* = .50, *p* = .022) and emotion regulation strategy (*b* = .49, *SE* = .24, *p* = .037). Simple slope analysis showed that the association between disgust and CRC screening intention was significant only for younger adults (*b* = −0.37, *SE* = .80, *p* < .001) compared to older adults (*b* = −0.09, *SE* = .07, *p* = .205), and only for those participants who preferred a situation selection strategy (*b* = −0.32, *SE* = .08, *p* < .001) rather than a reappraisal strategy (*b* = −0.12, *SE* = .08, *p* = .128). The three-way interaction between disgust, age group and emotion regulation strategy was not significant (*b* = −0.25, *SE* = .14, *p* = .070). The second moderation analysis, including embarrassment as focal predictor, yielded no significant main effect of this emotion (*b* = −1.75, *SE* = .91, *p* = .055), age group (*b* = −1.10, *SE* = .79, *p* = .164) and emotion regulation strategy (*b* = −0.13, *SE* = .34, *p* = .710) on the outcome, nor any significant interaction (Embarrassment*Age group, *b* = 0.96, *SE* = .56, *p* = .086; Embarrassment*Emotion regulation strategy, *b* = 0.30, *SE* = .24, *p* = .225; three-way interaction, *b* = −0.17, *SE* = .15, *p* = .241). The third moderation analysis, with fear of the outcome, yielded no significant main effect (Fear, *b* = 0.45, *SE* = .59, *p* = .446; Age group, *b* = 0.44, *SE* = .97, *p* = .649; Emotion regulation strategy, *b* = 0.60, *SE* = .43, *p* = .158) nor interaction (Fear*Age group, *b* = −0.18, *SE* = .38, *p* = .644; Fear*Emotion regulation strategy, *b* = −0.19, *SE* = .17, *p* = .281; three-way interaction, *b* = 0.08, *SE* = .11, *p* = .456).

The messages had no effect on the emotional barriers to CRC screening (Supplementary Table 2).

| **Supplementary Table 1.** |  |  |  |
| --- | --- | --- | --- |
| *2x2x2 ANOVA. Effects on CRC screening intention* |  |  |  |
|  | *df* | *F* | *p* |
| Affective lever | 1 | 1.14 | .287 |
| Cognitive lever | 1 | 2.91 | .089 |
| Affective * Cognitive lever | 1 | 0.41 | .524 |
| Emotion Regulation Strategy | 1 | 2.26 | .133 |
| Affective lever * Emotion Regulation Strategy | 1 | 2.92 | .088 |
| Cognitive lever * Emotion Regulation Strategy | 1 | 0.01 | .531 |
| Affective lever * Cognitive lever * Emotion Regulation Strategy | 1 | 0.39 | .144 |

| **Supplementary Table 2.** | | | | | | | |  | | |  | |  | |  | |  | |  | |
| --- | --- | --- | --- | --- | --- | --- | --- | --- | --- | --- | --- | --- | --- | --- | --- | --- | --- | --- | --- | --- |
| *One-way ANOVA. Effects of the messages on disgust, embarrassment and fear* | | | | | | | | |  | | |  | |  | |  | |  | |  |
|  |  | Disgust | | Embarrassment | | Fear | | | |  |  |  |  |  |  |  |  |  |  |  |
|  | *df* | *F* | *p* | *F* | *p* | *F* | *p* | | |  |  |  |  |  |  |  |  |  |  |  |
| Message | 3 | 0.13 | .941 | 0.64 | .590 | 0.59 | .625 | | |  |  |  |  |  |  |  |  |  |  |  |

# Electronic Supplementary Material 3


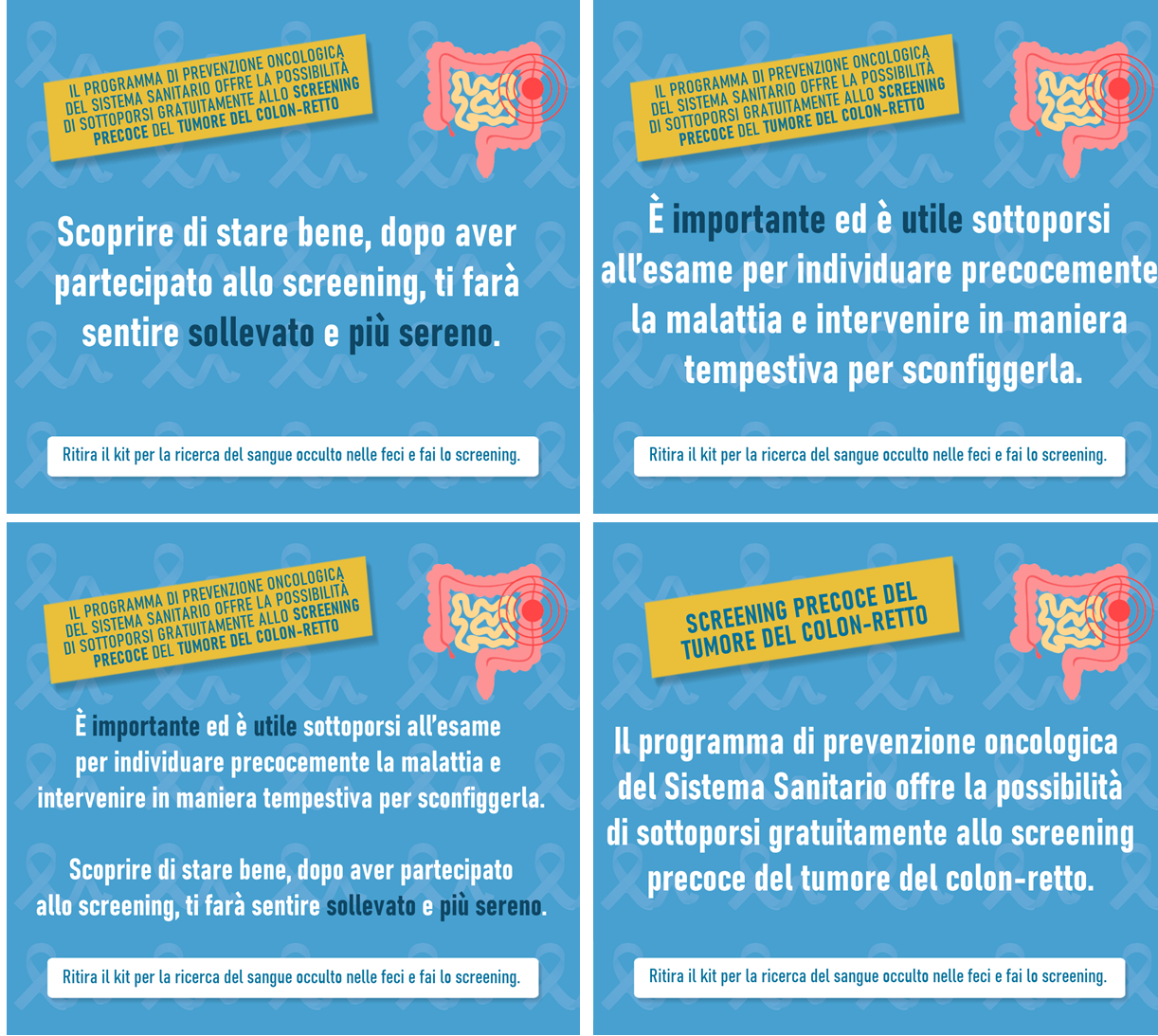


- Cognitive, affective and combined messange, yellow section: The National Healthcare system's cancer prevention program offers free early screening for colorectal cancer.
- Control message, yellow section: Colorectal cancer early screening.
- Cognitive lever: Attending CRC screening is *useful* and *important* for early detection and to improve your chances of defeating the disease.
- Affective lever: Finding out that you are healthy, after attending screening, will make you feel relieved and peaceful.
- Footprint (all messages): Pick up the kit for the fecal occult blood test and get screened.
